# Genome Sequences of Two *Leuconostoc pseudomesenteroides* Strains Isolated from Danish Dairy Starter Cultures

**DOI:** 10.1128/genomeA.00484-14

**Published:** 2014-06-05

**Authors:** T. B. Pedersen, W. P. Kot, L. H. Hansen, S. J. Sørensen, J. R. Broadbent, F. K. Vogensen, Y. Ardö

**Affiliations:** a Department of Food Science, Faculty of Science, University of Copenhagen, Copenhagen, Denmark; b Department of Biology, Faculty of Science, University of Copenhagen, Copenhagen, Denmark; c Department of Nutrition, Dietetics, and Food Sciences, Utah State University, Logan, Utah, USA; d Department of Environmental Science, Aarhus University, Roskilde, Denmark

## Abstract

The lactic acid bacterium *Leuconostoc pseudomesenteroides* can be found in mesophilic cheese starters, where it produces aromatic compounds from, e.g., citrate. Here, we present the draft genome sequences of two *L. pseudomesenteroides* strains isolated from traditional Danish cheese starters.

## GENOME ANNOUNCEMENT

Here, we present the draft genome sequences of two *Leuconostoc pseudomesenteroides* strains, PS12 and 1159, which were isolated from two different Danish mesophilic undefined cheese starters (1). *L. pseudomesenteroides* is a versatile organism that has been isolated from various food sources (2, 3). It produces diacetyl and contributes to the eye formation in Gouda type cheese via its heterofermetative metabolism and ability to degrade citrate (4). Currently, there are two publicly available *L. pseudomesenteroides* draft sequences: those of strain 4882, isolated from a French dairy starter culture (5), and strain 3652^T^, isolated from cane juice (6). Sequencing libraries were prepared using the Nextera XT kit (Illumina, USA), according to the manufacturer’s recommendations, followed by sequencing as a part of the flowcell, as 2 × 250-base paired-end reads using the Illumina MiSeq (Illumina, USA) technology. The reads were trimmed and assembled with the CLC Genomics Workbench 6.5.1 (CLC bio, Denmark). The resulting contigs were annotated using the RAST server (7). The two strains have similar sizes and genomic features (Table 1) and share a number of conserved functions, including genes for central carbohydrate metabolism and protein degradation. Both strains also contain clustered regularly interspaced short palindromic repeat (CRISPR) elements, which were also found in *L. pseudomesenteroides* strain 4882 but not in strain 3652^T^. The finding that the main differences between the two genomes were coding sequences (CDS) for CRISPR elements and different phage genes indicates a prominent influence of phage exposure on the adaptation of these strains in dairy environments. Future work with the four genomes will give more insight on the evolution and adaptation of *L. pseudomesenteroides* to different environments.

**TABLE 1:** General features for strains 1159 and PS12

Feature	*L. pseudomesenteroides* strain:	
	1159	PS12
Genome size (bp)	2,038,943	1,935,842
No. of open reading frames	2,109	1,964
G+C content (%)	38.9	39.0
No. of RNA genes	48	47
No. of contigs	100	91
Coverage (×)	100	380

### Nucleotide sequence accession numbers.

The whole-genome shotgun projects for *L. pseudomesenteroides* strains 1159 and PS12 have been deposited at DDBJ/EMBL/GenBank under the accession no. JAUI00000000 and JDVA00000000, respectively. The versions described in this paper are JAUJ01000000 and JDVA01000000, respectively.
